# Similarities and differences between stakeholders’ opinions on using Health Technology Assessment (HTA) information across five European countries: results from the EQUIPT survey

**DOI:** 10.1186/s12961-016-0110-7

**Published:** 2016-05-26

**Authors:** Zoltan Vokó, Kei Long Cheung, Judit Józwiak-Hagymásy, Silke Wolfenstetter, Teresa Jones, Celia Muñoz, Silvia M.A.A. Evers, Mickaël Hiligsmann, Hein de Vries, Subhash Pokhrel, On behalf of the EQUIPT Study Group

**Affiliations:** Department of Health Policy & Health Economics, Faculty of Social Sciences, Eötvös Loránd University, 1117 Budapest, Pázmány Péter sétány 1/a, Hungary; Syreon Research Institute, 1142 Budapest, Mexikói út 65/A, Hungary; Caphri School of Public Health and Primary Care, Health Services Research, Maastricht University, Duboisdomein 30, 6229 GT Maastricht, The Netherlands; Helmholtz Zentrum München, Ingolstädter Landstr. 1, 85764 Neuherberg, Germany; Health Economics Research Group, Brunel University London, Uxbridge, UB8 3PH United Kingdom; Centre for Research in Health and Economics, Pompeu Fabra University, Ramon Trias Fargas 25-27, 08005 Barcelona, Spain; Caphri School of Public Health and Primary Care, Health Promotion, Maastricht University, POB 616, 6200 MD Maastricht, The Netherlands

**Keywords:** Smoking cessation, EQUIPT, Stakeholder engagement, Return on investment, Transferability of economic evidence

## Abstract

**Background:**

The European-study on Quantifying Utility of Investment in Protection from Tobacco (EQUIPT) project aimed to study transferability of economic evidence by co-creating the Tobacco Return On Investment (ROI) tool, previously developed in the United Kingdom, for four sample countries (Germany, Hungary, Spain and the Netherlands). The EQUIPT tool provides policymakers and stakeholders with customized information about the economic and wider returns on the investment in evidence-based tobacco control, including smoking cessation interventions. A Stakeholder Interview Survey was developed to engage with the stakeholders in early phases of the development and country adaptation of the ROI tool. The survey assessed stakeholders’ information needs, awareness about underlying principles used in economic analyses, opinion about the importance, effectiveness and cost-effectiveness of tobacco control interventions, and willingness to use a Health Technology Assessment (HTA) tool such as the ROI tool.

**Methods:**

A cross sectional study using a mixed method approach was conducted among participating stakeholders in the sample countries and the United Kingdom. The individual questionnaire contained open-ended questions as well as single choice and 7- or 3-point Likert-scale questions. The results corresponding to the priority and needs assessment and to the awareness of stakeholders about underlying principles used in economic analysis are analysed by country and stakeholder categories.

**Results:**

Stakeholders considered it important that the decisions on the investments in tobacco control interventions should be supported by scientific evidence, including prevalence of smoking, cost of smoking, quality of life, mortality due to smoking, and effectiveness, cost-effectiveness and budget impact of smoking cessation interventions. The proposed ROI tool was required to provide this granularity of information. The majority of the stakeholders were aware of the general principles of economic analyses used in decision making contexts but they did not appear to have in-depth knowledge about specific technical details. Generally, stakeholders’ answers showed larger variability by country than by stakeholder category.

**Conclusions:**

Stakeholders across different European countries viewed the use of HTA evidence to be an important factor in their decision-making process. Further, they considered themselves to be capable of interpreting the results from a ROI tool and were highly motivated to use it.

**Electronic supplementary material:**

The online version of this article (doi:10.1186/s12961-016-0110-7) contains supplementary material, which is available to authorized users.

## Background

Tobacco smoking is one of the most serious public health problems both in Europe and worldwide. At the global level, the burden of disease due to tobacco smoking, including second-hand smoke, in 2010 was found to be 6.3 million deaths and 6.3% of disability-adjusted life years [1]. WHO estimated the percentage of deaths attributable to tobacco in Europe in 2004 as 25% of all deaths in men and 7% of all deaths in women [2]. The costs of smoking in Europe were estimated at just above 1% of the European Union Gross Domestic Product in 2000 [3].

In 2008, WHO identified six evidence-based tobacco control measures (the MPOWER measures) [4] that are the most effective in reducing tobacco use: (1) monitor tobacco use and prevention policies; (2) protect people from tobacco smoke; (3) offer help to quit tobacco use; (4) warn people about the dangers of tobacco; (5) enforce bans on tobacco advertising, promotion and sponsorship; and (6) raise taxes on tobacco. Recent research comparing the effects of the MPOWER measures has found an overall decrease in the prevalence of smoking but cross-country variations in these effects [5].

There is evidence on the cost-effectiveness of smoking cessation interventions and wider tobacco control [6, 7]. The main objective of such a health technology assessment (HTA) is to improve decision making about the adoption and diffusion of health technologies. However, in practice, it is not clear which HTA findings impact upon decision making [8]. As scarce resources are invested in HTA studies, it is important to maximize benefits from HTA activities. Koopmanschap et al. [9] argued that HTA research as a field is too young to understand how policymakers handle the multidimensional information of HTA studies. However, insights into what criteria (and under what conditions) decision makers in healthcare priority settings deem important may promote transparency of the decision-making procedure and enable researchers to collect relevant decision-support data [9].

Research on specific tobacco control interventions which is needed to make evidence-based decisions is disproportionate across Europe. While robust research evaluating effectiveness and cost-effectiveness of tobacco control policies, strategies, activities and interventions are on the rise in Western Europe, Central and Eastern European (CEE) countries do not have enough resources to perform effectiveness evaluation. One way to address this disparity is to transfer already established evidence to decision-making policies of the CEE countries. The European-study on Quantifying Utility of Investment in Protection from Tobacco (EQUIPT) aims to address this important gap.

EQUIPT was developed around evidence transferability and diffusion of innovation theory [10, 11]. The aim of this project was to study transferability of economic evidence by co-creating a new Tobacco Return On Investment (ROI) tool, adapted from a tool previously developed in England [7, 12], for five European member states (Germany, Hungary, Spain, the Netherlands and the United Kingdom), and provide policymakers and stakeholders with customized information about the economic and wider returns that investment in evidence-based tobacco control, including smoking cessation, agendas can generate. This sample represents the whole European continent, including several cultural, social and economic habits and traditions. The choice of countries facilitates the transferability of the ROI tool’s results to other countries not considered in EQUIPT [13]. The ROI tool, which is a Markov state transition model, is able to compare the effectiveness and costs of tobacco control strategies both within and across several European Union countries.

To inform the co-creation of a European decision-support tool, a stakeholder interview survey was conducted. The purpose of the survey, conducted by the sample country-specific researchers, was to engage stakeholders in an early phase of the development and country adaptation of the ROI tool in order to facilitate its later use [13]. EQUIPT considered ‘stakeholder engagement’ as a tool to understand the issues that are important to transfer evidence. Research that has potential to directly feed into policymaking is often produced without engaging stakeholders in the research process [14]. However, the importance of stakeholder engagement in research is increasingly recognised [15] and several reviews indicate that the interaction between health researchers and potential users in policy and managerial roles is a key factor associated with the impact of the research [16–19].

In this paper, we describe the EQUIPT Stakeholder Interview Survey with the following three aims: (1) to investigate the criteria stemming from HTA studies that are deemed most important for reimbursement decisions concerning tobacco control, (2) to examine whether there are any country differences, and (3) to examine whether there are differences among different types of stakeholder categories.

## Methods

### Scope of the survey

A cross-sectional mixed method study, in which qualitative and quantitative research techniques were combined, was conducted among stakeholders from the five European countries (Germany, Hungary, Spain, the Netherlands and the United Kingdom). The questionnaire used in the stakeholder interviews was developed by EQUIPT project partners through an intensive deliberation and partly based on the Integrated-Change (I-Change) Model and other relevant constructs related to the adoption of innovations [11, 13, 20, 21].

The survey’s results, corresponding to the priority and needs assessment and to the awareness of stakeholders about the principles of health economic analysis, are presented in this paper by country and stakeholder categories. Stakeholders’ views on the possible advantages and disadvantages of using the ROI tool, internal and external facilitators, and barriers of the use by intenders and non-intenders were analysed and reported in the framework of the I-Change model by Cheung et al. [21]. The availability and the importance of the smoking cessation interventions used in the sample countries will be reported separately, elsewhere.

### Development of the questionnaire

The first English version of the questionnaire was tested and adapted further by taking into consideration the results of the pilot studies. All country-specific interviewers participated in pre-interview training, in which they acquired knowledge on interviewing methodology (what they should pay attention to during the interview, how to ask, how to motivate the respondents, what is the meaning of each question, etc.). The final version of the questionnaire was translated by native language speakers. Translated versions were pilot tested again in each country, and then finalized after further adaptations. The interviewers were aided by a survey handbook as well.

### Questionnaire

The first part of the questionnaire assessed the stakeholders’ awareness of basic principles underlying an economic analysis (e.g. what incremental cost or willingness to pay might refer to), and about the importance, effectiveness and cost-effectiveness of smoking cessation interventions in general (e.g. why it is important to use effective and cost-effective interventions). Then, a video presentation on the existing United Kingdom version of the Tobacco ROI tool was shown, followed by questions about the types of information required from the EQUIPT ROI tool. Opinion about the possible advantages (e.g. the tool provides up-to-date information, relevant outcomes and sufficient scientific support for decision making, etc.) and disadvantages (e.g. the tool requires excessive data input and it is time consuming to use, etc.) of using the ROI tool were also assessed together with potential facilitators and barriers of use (e.g. my boss, reimbursement agencies or ministry of health will support me in using this tool, etc.). Additionally, self-efficacy and intention to use was assessed. Finally, the respondent’s awareness of the availability of smoking cessation interventions in the target country and their importance to the respondent were asked. The main categories of interventions were pharmacological (e.g. nicotine replacement therapy, bupropion, varinicline), behavioural (e.g. brief advice on smoking cessation given during one general practitioner consultation, group counselling by specially trained professionals, mobile phone-based interventions, etc.), combined (combination of pharmacological and behavioural interventions), non-conventional (e.g. acupuncture, herbs, homeopathy, etc.) and population-level preventive measures (e.g. advertising restrictions/bans, restrictions on smoking in workplaces and public places, tax increase, etc.) (See Additional file 1 – EQUIPT stakeholder interview survey questionnaire).

Open-ended questions as well as single choice and Likert-scale questions were asked where appropriate. For example, the questions on the stakeholders’ views on their intention to use a tool in decision making was designed as a 7-point Likert-scale (1 = ‘strongly disagree’ and 7 = ‘strongly agree’).

### Stakeholder categories

Five major categories of stakeholders were defined: (1) decision makers; (2) purchasers of services/pharma products; (3) professional service providers; (4) evidence generators; and (5) advocates of health promotion. The EQUIPT team members defined the five stakeholder categories and specified the roles and positions of each stakeholder category together. The definitions for each category and examples are shown in Table 1. Then, the interviewers had to recruit stakeholders in their own countries in such a way that a minimum of three stakeholders had to be invited from each stakeholder category. In total, 167 stakeholders were contacted, 93 of whom were willing to participate in the survey. An introductory mail/email was sent to the stakeholders introducing the EQUIPT project and the Tobacco ROI tool. Participants accepting the invitation then received an information sheet and a consent form for completion. Data were handled anonymously and treated confidentially as per the conditions underlying ethics approval.

**Table 1:** Types of stakeholders

Stakeholder category	Role	Position (examples)
Decision maker	Decision maker at the level of the national health policy	Member of the health committee of theParliament or high level decision maker, officer in the Ministry of Health
	Decision maker in the central level of public health services	Chief medical officer or other high level decision maker in the public health service
	Decision maker at the regional level in the area of health	Member of the health committee of the regional self-government or health officer at the regional health authority
	Local decision maker in a larger city in the area of health	Head or member of the health committee of the local authority
	Local decision maker in a small community (village) in the area of health	Member of the self-government or officer in the municipality office responsible for health issues
	Local decision maker in the public health service	Director of the local office of the public health service
Purchasers of services/pharma products	Decision maker involved in coverage decision of health services	High level decision maker at the National Health Insurance Fund, health insurance company depending on the health service of the country
	Decision maker involved in coverage decision of pharmaceuticals	High level decision maker at the National Health Insurance Fund, health insurance company depending on the health service of the country
Professionals/service providers	Coordinator of the local health program	Officer in the local health office of the government or the municipality
	Key opinion leader physician on smoking cessation	Director of a professional organisation of pulmonology or other relevant discipline or a large smoking cessation service
	Physician/psychologist with an overview on smoking cessation	Person working for smoking cessation hot line
Evidence generators (e.g. researchers) whose work informs policy/procurement/delivery of services	Health technology assessment (HTA) professional involved in the reimbursement procedure	Officer in the local HTA office
	Head of the local focal point of tobacco control	
	Public health expert	Director of a public health professional association or body, or relevant academic department
	Researcher in the field of the economy of smoking	Researcher with documented publication in the area of the economy of smoking (burden of disease, budget impact or cost-effectiveness of interventions, etc.)
	Expert in the area of healthcare costing	Researcher with documented publication in the area of healthcare costing or an expert working at the National Health Insurance Fund, health insurance company depending on the health service of the country, who is involved in professional work related to financing health services/pharma products
Advocates of health promotion	Leader of an NGO	Director or other leader of an NGO focusing on tobacco control
	Leader of a patient organisation	Leader of a patient organisation of cancer/chronic obstructive pulmonary disease patients

### Interview, data collection and analysis

The individual – face-to-face, telephone or Skype – interviews with stakeholders were conducted in the five countries (Germany, Hungary, Spain, the Netherlands and the United Kingdom) between April and July 2014. The interviews, with a duration of approximately 30 minutes, were audio recorded. The answers were recorded in a pre-prepared, unified data entry Microsoft Excel file. Double data entry was used to guarantee a high level of data quality. After the interview, an email was sent to all interviewees thanking them for their participation and providing a web link where more information about the EQUIPT project and the ROI tool could be found.

SPSS Statistics software package was used for data analysis. At first, we calculated the minimum and maximum values of the variables, the mean scores and the standard deviations for each of the questions at an aggregated level. Then, we calculated these measures by stakeholder category and by country. We used Kruskal–Wallis ANOVA test to study whether the distribution of the answers were different by country or by stakeholder category. This analytical technique was deemed adequate for the purpose of this study (i.e. high level comparative account), bearing in mind that each domain covered by the survey would be subject to separate future analyses.

## Results

A total of 93 respondents agreed to participate in the stakeholder interview survey, of whom 62% were male and 30.1% were Dutch, 19.4% Spanish, 18.3% German, 17.2% Hungarian, and 15.1% British. The overall response rate was 56.9% (93/167), while it was 94.1% in Hungary, 85.0% in Germany, 60.0% in Spain, 41.8% in the Netherlands, and 40.0% in the United Kingdom. Most (66.67%) of the interviews were face-to-face, whereas 32.26% were by telephone and only 1.08% were conducted via Skype. The breakdown of the different stakeholder categories was as follows: decision makers 31.2%, evidence generators 26.9%, professional service providers 19.4%, advocates of health promotion 15.1%, and purchaser of services/pharma products 7.5% [21].

### Awareness of principles underlying economic analyses in public health

The majority of the stakeholders were familiar of basic principles such as incremental cost, cost-effectiveness and perspectives of health economic analysis (Table 2). The majority of the interviewees were aware that a cheap intervention is not necessarily cost-effective, and that an intervention with higher societal costs than the regular care can be cost-effective. The respondents were least familiar with ‘willingness to pay’. There was a statistically significant difference between the countries in respondents understanding of ‘incremental costs’. The mean score showed reasonable agreement with the correctly phrased statement in Hungary, Germany and Spain. The Dutch and British responders, however, were uncertain about it (Table 2). The responses to the statement related to a technical detail of health economics – e.g. inclusion of the indirect costs in analyses from healthcare payer perspective – showed largest variability within countries, and the country means reflected uncertainty. Unsurprisingly, evidence generators were the most familiar group in terms of awareness of economic concepts (Table 3).

**Table 2:** Awareness of stakeholders about basic health economic principles; level of agreement with statements by country (scale responses: 1 = strongly disagree; 7 = strongly agree)

	Netherlands		Hungary		Germany		Spain		United Kingdom		Total		Kruskal–Wallis test
Statements	n	Mean (SD)	n	Mean (SD)	n	Mean (SD)	n	Mean (SD)	n	Mean (SD)	n	Mean (SD)	*P* value
‘Incremental costs’ means by how much the studied intervention itself costs more or less than the comparator intervention	28	4.79 (1.93)	10	6.60 (0.52)	13	5.62 (1.12)	16	6.25 (0.78)	14	3.71 (1.86)	81	5.25 (1.76)	<10^–3^
When an intervention in itself is cheap, it is always cost-effective compared to another intervention	28	1.96 (1.40)	16	1.50 (1.42)	17	1.94 (1.75)	18	2.28 (2.02)	14	1.57 (0.76)	93	1.88 (1.53)	0.35
‘Willingness to pay’ means how much a society is willing to pay for a quality-adjusted life year	28	4.86 (1.88)	11	4.45 (2.07)	14	3.71 (1.82)	13	4.92 (1.89)	14	3.86 (1.46)	80	4.44 (1.86)	0.14
My intervention can be cost-effective compared to another intervention, even when its societal costs are higher than the regular care	28	5.82 (1.63)	15	4.93 (2.60)	17	5.18 (1.82)	17	5.76 (1.44)	14	4.86 (1.79)	91	5.40 (1.83)	0.23
From a healthcare payer perspective indirect costs in full (such as productivity losses) are included	28	3.36 (2.41)	15	2.40 (1.81)	16	2.63 (2.06)	14	2.86 (1.99)	12	3.75 (1.87)	85	3.02 (2.11)	0.28

SD, Standard deviation

**Table 3:** Awareness of stakeholders about basic health economic principles; level of agreement with statements by stakeholder category (scale responses: 1 = strongly disagree; 7 = strongly agree)

	Decision-maker		Purchaser of services/pharma products		Professional service provider		Evidence generator		Advocate of health promotion		Total		Kruskal–Wallis test
Statements	n	Mean (SD)	n	Mean (SD)	n	Mean (SD)	n	Mean (SD)	n	Mean (SD)	n	Mean (SD)	*P* value
‘Incremental costs’ means by how much the studied intervention itself costs more or less than the comparator intervention	26	4.50 (1.77)	7	5.43 (1.90)	15	5.60 (1.64)	22	5.95 (1.50)	11	5.00 (1.84)	81	5.25 (1.76)	0.027
When an intervention in itself is cheap, it is always cost-effective compared to another intervention	29	1.76 (1.15)	7	2.29 (2.22)	18	2.89 (2.14)	25	1.32 (0.75)	14	1.64 (1.50)	93	1.88 (1.53)	0.027
‘Willingness to pay’ means how much a society is willing to pay for a quality-adjusted life year	26	4.19 (1.98)	7	3.86 (2.12)	15	4.87 (0.83)	22	4.64 (2.22)	10	4.40 (1.71)	80	4.44 (1.86)	0.73
My intervention can be cost-effective compared to another intervention, even when its societal costs are higher than the regular care	28	4.93 (1.84)	7	5.43 (2.07)	18	5.50 (1.69)	25	5.80 (1.92)	13	5.46 (1.71)	91	5.40 (1.83)	0.24
From a healthcare payer perspective indirect costs in full (such as 7productivity losses) are included	27	3.30 (2.15)	7	3.14 (2.04)	18	2.94 (2.16)	23	2.96 (2.18)	10	2.50 (2.12)	85	3.02 (2.11)	0.76

SD, Standard deviation

### Importance, efficacy and cost-effectiveness of smoking cessation interventions

All stakeholders across the countries strongly agreed that mortality and societal costs of smoking were high and thought that its epidemic was severe (Table 4). Respondents from the Netherlands, Hungary and Germany were uncertain about effectiveness and cost-effectiveness of the smoking cessation interventions, while the interviewees in Spain and the United Kingdom mostly agreed with the statements that these interventions are effective and cost-effective. The differences were statistically significant both for effectiveness and cost-effectiveness. Although the level of agreement varied significantly by country, the interviewees’ general opinion was that it was unacceptable that smoking cessation interventions are used without knowing their efficacy and their cost-effectiveness. There were no large differences in these answers by stakeholder category (Table 5). Professional service providers agreed the most with the effectiveness and cost-effectiveness of smoking cessation interventions, and purchasers of services agreed the most with the statement that it is unacceptable that we use smoking cessation interventions without knowing their efficacy. This was the only statement in this block where there was a statistically significant difference in the distribution of the answers by stakeholder category.

**Table 4:** Opinion about the importance, effectiveness and cost-effectiveness of smoking cessation interventions by country (scale responses: 1 = strongly disagree; 7 = strongly agree)

	Netherlands		Hungary		Germany		Spain		United Kingdom		Total		Kruskal–Wallis test
Statements	n	Mean (SD)	n	Mean (SD)	n	Mean (SD)	n	Mean (SD)	n	Mean (SD)	n	Mean (SD)	*P* value
The smoking epidemic is not severe in my country	28	2.00 (1.39)	16	1.44 (0.89)	17	1.82 (1.13)	18	1.50 (0.86)	14	2.50 (1.65)	93	1.85 (1.251)	0.10
Most smoking cessation interventions are effective	28	3.79 (1.52)	16	2.63 (1.31)	17	2.71 (0.85)	18	4.67 (1.75)	14	4.79 (1.42)	93	3.71 (1.639)	< 10^–3^
Most smoking cessation interventions are cost-effective	28	4.11 (1.91)	14	2.64 (1.48)	17	3.18 (1.67)	16	4.75 (1.88)	14	5.71 (1.27)	89	4.07 (1.941)	< 10^–3^
It is important to use smoking cessation interventions because smoking kills a lot of people	28	6.46 (0.79)	16	6.88 (0.50)	17	5.82 (1.51)	18	6.83 (0.51)	14	6.71 (0.47)	93	6.53 (0.916)	0.009
It is important to use smoking cessation interventions because smoking costs a lot for the society	28	6.14 (1.01)	16	6.75 (0.78)	17	5.76 (1.56)	18	6.67 (0.69)	14	6.43 (0.76)	93	6.32 (1.055)	0.032
It is unacceptable that we use smoking cessation interventions without knowing their efficacy	27	5.89 (1.05)	16	6.88 (0.34)	17	5.65 (1.62)	17	5.41 (1.54)	14	5.43 (1.22)	91	5.86 (1.304)	0.003
It is unacceptable that we use smoking cessation interventions without knowing their cost-effectiveness	27	5.04 (1.56)	16	6.00 (1.41)	17	5.12 (1.73)	18	4.50 (1.79)	14	5.07 (1.39)	92	5.12 (1.623)	0.092

SD, Standard deviation

**Table 5:** Opinion about the importance, effectiveness and cost-effectiveness of smoking cessation interventions by stakeholder category (scale responses: 1 = strongly disagree; 7 = strongly agree)

	Decision-maker		Purchaser of services/pharma products		Professional service provider		Evidence generator		Advocate of health promotion		Total		Kruskal–Wallis test
Statements	n	Mean (SD)	n	Mean (SD)	n	Mean (SD)	n	Mean (SD)	n	Mean (SD)	n	Mean (SD)	*P* value
The smoking epidemic is not severe in my country	29	1.76 (1.22)	7	2.00 (0.58)	18	1.78 (1.44)	25	2.04 (1.40)	14	1.71 (1.14)	93	1.85 (1.25)	0.42
Most smoking cessation interventions are effective	29	3.72 (1.67)	7	3.57 (1.27)	18	4.17 (1.58)	25	3.56 (1.69)	14	3.43 (1.83)	93	3.71 (1.64)	0.72
Most smoking cessation interventions are cost-effective	27	4.26 (1.95)	6	3.83 (1.47)	18	4.39 (2.06)	24	3.71 (2.14)	14	4.00 (1.71)	89	4.07 (1.94)	0.80
It is important to use smoking cessation interventions because smoking kills a lot of people	29	6.59 (0.83)	7	6.14 (0.90)	18	6.50 (1.34)	25	6.60 (0.76)	14	6.50 (0.76)	93	6.53 (0.92)	0.42
It is important to use smoking cessation interventions because smoking costs a lot for society	29	6.62 (0.78)	7	5.57 (1.27)	18	6.28 (1.27)	25	6.28 (0.98)	14	6.21 (1.19)	93	6.32 (1.06)	0.15
It is unacceptable that we use smoking cessation interventions without knowing their efficacy	28	5.75 (1.14)	7	6.71 (0.49)	18	5.33 (1.68)	25	6.24 (0.88)	13	5.62 (1.71)	91	5.86 (1.30)	0.13
It is unacceptable that we use smoking cessation interventions without knowing their cost-effectiveness	29	5.17 (1.61)	7	6.00 (0.58)	18	4.06 (1.83)	25	5.76 (1.48)	13	4.77 (1.24)	92	5.12 (1.62)	0.009

SD, Standard deviation

### HTA information needed by decision-makers from the ROI tool

In general, all the listed HTA information, including prevalence of smoking, cost of smoking, quality of life, mortality due to smoking, effectiveness of smoking cessation interventions (such as quit and relapse rate), cost-effectiveness data comparing the cost of smoking cessation interventions with its health and wider benefits and budget impact reflecting financial outcomes specifically at organisational level, was considered very important and the stakeholders wanted the ROI tool to provide all of this information (Table 6). Information on the cost-effectiveness of smoking cessation interventions was found to be the most important health technology assessment (HTA) information in most countries (except in Hungary and Germany, where the costs of smoking had the largest mean score; Table 6). In Hungary, the burden of disease due to smoking measures (prevalence, costs and mortality of smoking, effect on quality of life) had higher mean scores than in any of the other participating countries. Nevertheless, health economic measures were not undervalued in Hungary compared to the other countries. In countries where cost-effectiveness was considered to be the most important of the HTA data to be included, it was followed by the cost of smoking in the United Kingdom, effectiveness of smoking cessation interventions in the Netherlands and budget impact in Spain. The importance of information on costs of smoking and smoking-attributable mortality showed statistically significant variations by country, both receiving the highest score in Hungary and the lowest in Spain, for costs of smoking and in Germany for smoking-attributable mortality. The distribution of the answers by the stakeholder category did not show a statistically significant variation in case of any HTA information (Table 7). In most stakeholder categories, except for professional service providers, cost-effectiveness was considered to be the most important information.

**Table 6:** Health technology assessment information deemed to be important by stakeholders by country (scale responses: 1 = strongly disagree; 7 = strongly agree)

	Netherlands		Hungary		Germany		Spain		United Kingdom		Total		Kruskal–Wallis test
Statements	n	Mean (SD)	n	Mean (SD)	n	Mean (SD)	n	Mean (SD)	n	Mean (SD)	n	Mean (SD)	*P* value
Prevalence of smoking	28	5.89 (1.34)	16	6.38 (1.41)	17	5.82 (1.55)	18	5.78 (1.96)	14	5.64 (2.41)	93	5.90 (1.69)	0.38
Costs of smoking	28	6.29 (0.85)	16	7.00 (0.00)	17	6.53 (0.62)	18	6.28 (1.41)	14	6.71 (1.07)	93	6.52 (0.94)	0.002
Quality of life	28	5.79 (1.32)	16	6.13 (1.26)	17	5.41 (1.87)	18	6.06 (1.39)	14	5.86 (1.10)	93	5.84 (1.40)	0.63
Mortality due to smoking	28	6.11 (1.26)	16	6.75 (1.00)	17	5.88 (1.93)	18	6.33 (1.03)	14	6.64 (1.34)	93	6.30 (1.35)	0.010
Effectiveness of smoking cessation interventions (such as quit and relapse rates)	28	6.39 (1.20)	16	6.50 (1.27)	17	6.18 (1.67)	18	6.39 (1.42)	14	6.71 (1.07)	93	6.42 (1.31)	0.35
Cost-effectiveness data comparing the cost of smoking cessation interventions with its health and wider benefits	28	6.43 (0.88)	16	6.69 (0.48)	17	6.18 (1.02)	18	6.78 (0.43)	14	6.86 (0.36)	93	6.56 (0.74)	0.093
Budget impact reflecting financialoutcomes specifically at organisational level	28	6.07 (1.25)	16	6.69 (0.60)	17	6.00 (1.37)	18	6.67 (0.49)	14	6.00 (1.47)	93	6.27 (1.13)	0.17

SD, Standard deviation

**Table 7:** Health technology assessment information deemed to be important by stakeholders by stakeholder category (scale responses: 1 = strongly disagree; 7 = strongly agree)

	Decision-maker		Purchaser of services/pharma products		Professional service provider		Evidence generator		Advocate of health promotion		Total		Kruskal–Wallis test
Statements	n	Mean (SD)	n	Mean (SD)	n	Mean (SD)	n	Mean (SD)	n	Mean (SD)	n	Mean (SD)	*P* value
Prevalence of smoking	29	5.66 (2.01)	7	6.71 (0.49)	18	6.33 (1.28)	25	5.84 (1.72)	14	5.57 (1.70)	93	5.90 (1.69)	0.43
Costs of smoking	29	6.55 (0.83)	7	6.71 (0.76)	18	6.61 (0.78)	25	6.36 (1.32)	14	6.50 (0.65)	93	6.52 (0.94)	0.81
Quality of life	29	6.17 (0.85)	7	6.14 (1.22)	18	5.61 (1.58)	25	5.64 (1.66)	14	5.64 (1.69)	93	5.84 (1.40)	0.76
Mortality due to smoking	29	6.59 (0.98)	7	6.57 (0.79)	18	6.33 (1.03)	25	6.20 (1.50)	14	5.71 (2.09)	93	6.30 (1.35)	0.68
Effectiveness of smoking cessation interventions (such as quit and relapse rates)	29	6.59 (0.83)	7	6.71 (0.76)	18	6.56 (1.20)	25	6.12 (1.76)	14	6.29 (1.59)	93	6.42 (1.31)	0.76
Cost-effectiveness data comparing the cost of smoking cessation interventions with its health and wider benefits	29	6.66 (0.48)	7	6.86 (0.38)	18	6.33 (0.97)	25	6.52 (0.82)	14	6.57 (0.85)	93	6.56 (0.74)	0.65
Budget impact reflecting financial outcomes specifically at organisational level	29	6.17 (1.39)	7	6.57 (0.56)	18	6.33 (0.97)	25	6.20 (1.19)	14	6.36 (0.93)	93	6.27 (1.13)	0.99

SD, Standard deviation

The vast majority (81%) of the stakeholders proposed other types of evidence in the open-ended questions than those provided by the ROI tools, which they considered important in the decision-making process on the implementation of tobacco control measures and smoking cessation interventions. Their suggestions varied greatly, the most frequent propositions were information about absenteeism, days off work, more details about the smoking cessation interventions (e.g. effectiveness, accessibility), cost, effect of smoking-related diseases on quality of life, indirect costs, more detailed healthcare utilization data popularity of a smoking cessation programme among smokers, and results of target group analysis.

### Intention to use the ROI tool

Stakeholders from all sample countries expressed their willingness to use a tool such as the Tobacco ROI tool (Table 8). The Hungarian stakeholders were the most committed to its use, while the Dutch and German interviewees were least interested. Respondents from Hungary and Spain wanted to use the tool basically as soon as possible, while in the United Kingdom and Germany they were more likely to introduce it in a year, and the Dutch were uncertain about the timeframe of its utilisation. The respondents almost unanimously expressed that they would have liked to have more information about the Tobacco ROI tool. The answers by the stakeholder category were more homogeneous. Nevertheless, the purchasers of services and pharma products were those who most intended to use the tool, and the advocates of health promotion were the least committed stakeholder group (Table 9).

**Table 8:** Stakeholders’ intention to use a tool such as the Tobacco ROI tool by country (scale responses: 1 = strongly disagree; 7 = strongly agree)

	Netherlands		Hungary		Germany		Spain		United Kingdom		Total		Kruskal–Wallis test
Statements	n	Mean (SD)	n	Mean (SD)	n	Mean (SD)	n	Mean (SD)	n	Mean (SD)	n	Mean (SD)	*P* value
I have the intention to use a tool such as the Tobacco ROI tool	28	4.64 (1.93)	16	6.88 (0.50)	17	4.47 (1.97)	17	6.29 (0.99)	14	5.57 (1.83)	92	5.45 (1.83)	< 10^–3^
I have the intention to use a tool such as the Tobacco ROI tool within the next month	28	3.18 (2.00)	16	6.31 (1.45)	17	3.00 (2.12)	16	6.38 (0.89)	14	4.14 (2.21)	91	4.41 (2.32)	< 10^–3^
I have the intention to use a tool such as the Tobacco ROI tool within the next 6 months	28	3.89 (2.13)	16	6.50 (1.10)	17	3.35 (2.23)	16	5.44 (1.75)	14	5.50 (1.65)	91	4.77 (2.16)	< 10^–3^
I have the intention to use a tool such as the Tobacco ROI tool within the next year	28	4.54 (2.13)	16	6.75 (0.58)	17	4.65 (1.94)	16	4.44 (1.97)	14	5.86 (1.66)	91	5.13 (1.98)	< 10^–3^
I would like to have more information about the Tobacco ROI tool	28	6.00 (1.54)	16	6.44 (1.32)	17	6.06 (1.56)	17	6.65 (0.79)	14	6.50 (0.76)	92	6.28 (1.30)	0.31

SD, Standard deviation

**Table 9:** Stakeholders’ intention to use a tool such as the Tobacco ROI tool by stakeholder category (scale responses: 1 = strongly disagree; 7 = strongly agree)

	Decision-maker		Purchaser of services/pharma products		Professional service provider		Evidence generator		Advocate of health promotion		Total		Kruskal–Wallis test
Statements	n	Mean (SD)	n	Mean (SD)	n	Mean (SD)	n	Mean (SD)	n	Mean (SD)	n	Mean (SD)	*P* value
I have the intention to use a tool such as the Tobacco ROI tool	29	5.59 (1.78)	7	5.71 (1.50)	18	5.44 (1.54)	24	5.42 (2.00)	14	5.07 (2.27)	92	5.45 (1.83)	0.97
I have the intention to use a tool such as the Tobacco ROI tool within the next month	29	4.90 (2.29)	7	3.57 (2.57)	17	5.06 (1.92)	24	3.92 (2.28)	14	3.86 (2.63)	91	4.41 (2.32)	0.36
I have the intention to use a tool such as the Tobacco ROI tool within the next 6 months	29	5.31 (2.12)	7	3.71 (2.06)	17	5.00 (2.06)	24	4.63 (2.06)	14	4.14 (2.48)	91	4.77 (2.16)	0.22
I have the intention to use a tool such as the Tobacco ROI tool within the next year	29	5.24 (2.12)	7	4.86 (2.12)	17	5.41 (1.73)	24	5.08 (2.08)	14	4.79 (1.93)	91	5.13 (1.98)	0.77
I would like to have more information about the Tobacco ROI tool	29	6.00 (1.79)	7	6.00 (1.83)	18	6.50 (0.62)	24	6.50 (0.98)	14	6.36 (0.84)	92	6.28 (1.30)	0.80

SD, Standard deviation

## Discussion

The present study examined stakeholders’ views on the use of the EQUIPT Tobacco ROI tool, a HTA-type decision support aid for tobacco control. We collected the views of stakeholders with various roles across five countries by a semi-structured survey, and analysed the data, including by stakeholder role and country, and reported the initial findings.

A number of implications emerge from this unique survey. Firstly, this study suggests that the stakeholders consider it important that decisions on investments in smoking cessation interventions should be supported by HTA evidence. Further, stakeholders believe that all the HTA information assessed (prevalence and cost of smoking, quality of life, mortality due to smoking, effectiveness and cost-effectiveness of smoking cessation interventions, and budget impact) is of great importance and want the ROI tool to provide them with this information. The majority of the stakeholders were familiar with basic health economic concepts, but did not have in-depth knowledge about the technical details. Overall, the Tobacco ROI tool was received positively by stakeholders; they were waiting for the final tool to be available for use, and most of the respondents would have liked to use a tool such as the Tobacco ROI tool within a year.

Secondly, when analysing the similarities and differences in views per stakeholder, the findings showed that most stakeholders agreed with most questions which were included in the questionnaire. They thought that using a tool such as the EQUIPT Tobacco ROI tool in decision-making was important. Nevertheless, the study found differences between stakeholders: professional service providers agreed the most with the statements that smoking cessation interventions were effective and cost-effective, while the purchasers of services thought mostly that it was unacceptable that smoking cessation interventions were used without their efficacy being known. Most of the stakeholders, except for professional service providers, believed that the most important HTA information was the cost-effectiveness of an intervention. In accordance with our expectations, advocates of health promotion intended to use a tool such as the ROI tool the least, likely due to the burden of disease influencing these stakeholders much more than the return on investment.

Thirdly, when analysing the similarities and differences in views per country, the greatest level of agreement existed for the statements that mortality and societal costs of smoking were high and its epidemic was severe. However, differences between countries were notable for most questions regarding the relative importance of different elements of HTA information.

The variation by stakeholder categories indicates that different skillsets may exist amongst the stakeholders of the included countries, and is in line with previous work by the National Institute for Health and Care Excellence (NICE) that found a range of people within England could be involved in decision making about investment in public health interventions [22]. This study corroborates that involving stakeholders early on in a research project increases the likelihood of the research findings being used in policymaking. Well thought-through stakeholder engagement is therefore seen as a ‘pathway to research impact’. Our results support the conclusion of the NICE study, which reported that a conversation between decision makers and health economic researchers should start at early stages of model development [22]. The differences found between the stakeholder categories indicated that we need consider differentiation of variant user levels (basic, moderate and advanced level) of a tool such as the ROI tool in the development process and to apply different outcome indicators by user level.

Generally, stakeholders’ answers showed larger variability by country than by stakeholder category. This is interesting and highlights the importance of collecting the views of stakeholders within each country as a step in the process of developing a transferable tool. Without these views, the usefulness of the Tobacco ROI tool beyond the country for which it was developed would be unknown. Previous research [23–25] had discussed the importance of context when considering transferability of economic evidence and this factor will inform ongoing work on the transferability of the EQUIPT Tobacco ROI tool [13].

Once transferability within the sample countries has been tested, we can consider further transferability of the ROI tool to other countries beyond the sample countries. This will enable countries with a smaller tobacco control budget, but with perhaps a greater need for tobacco control, to do so with more informed techniques. This, in turn, should help reduce tobacco use and thereby improve the health of their citizens and reduce the burden of disease due to smoking [4].

A limitation of our study is that the stakeholders cannot be considered as a representative sample of all stakeholders in the participating countries. Nevertheless, a range of major stakeholder categories were represented from each participating country and the final sample was the outcome of random responses from our sampling frame. Therefore, we believe that our results give a generalizable insight to the attitude and expectations of stakeholders in relation to the use of the Tobacco ROI tool. Another limitation of this research is that the interviewing method was not unified, most interviews were face-to-face, but in some cases these were conducted by telephone or Skype. In a face-to-face interview the uncertainty of the responders can be managed much better and the involvement in the survey can be more effective than in a telephone or Skype interview. Nevertheless, we think that the small number of telephone or Skype interviews do not endanger the credibility of the results.

Although HTA processes are based on internationally recognised methods, significant disparities have been observed to date in the recommendations made by HTA agencies among different countries due to the quality and type of evidence used [26]. Results of these studies vary in context and topics. Regarding tobacco control interventions, there has been no evidence about the types of information deemed important for coverage decision. A large number of attributes of interventions may be important in the decision-making process (e.g. disease severity, budget impact and costs per quality-adjusted life-year gained) [27–29]. Koopmanschap et al. [9] found that the most decisive decision criteria are severity of disease, costs per quality-adjusted life-year gained, individual health gain and the budget impact. Previous studies have suggested that cost-effectiveness is not the single and dominant concern [27, 28]. Our survey aimed to fill in this gap with collecting the types of information deemed to be important for decision to support the development and transferability of the ROI tool.

An important advantage of this survey is that it offers a unique dataset on the topic to fully analyse several aspects of stakeholder engagement in research. For example, there is a paucity of literature fully answering questions as to what determines a stakeholder’s likelihood of using economic evidence in decision making, what the level of agreement is within and between countries about what interventions are relevant to their settings, and whether the perceived ranking of what intervention is important to stakeholder actually aligns with the ranking indicated by published cost-effectiveness analyses. Most transferability work in economic evaluation appears to ignore this important pre-work involving stakeholder engagement [30]. In this respect, future in-depth analyses based on this dataset investigating the above questions would be highly welcome.

## Conclusions

This study shows that various types of stakeholders from different countries consider HTA evidence important in their decision-making process. They also consider themselves capable of interpreting the results from a return on investment tool, and they are generally mostly motivated to use a tool such as the ROI tool to aid their decision making. The survey findings serve as important input to the development and country adaptation of the ROI tool to ensure that the final product of the EQUIPT project would reflect the needs and expectations of the stakeholders.

## Abbreviations

CEE, Central and Eastern European; EQUIPT, European-study on Quantifying Utility of Investment in Protection from Tobacco; HTA, health technology assessment; I-Change Model, Integrated-Change Model; NICE, National Institute for Health and Care Excellence; ROI, return on investment

## Additional file

Additional file 1: EQUIPT stakeholder interview survey questionnaire. Description of data: The questionnaire used in the stakeholder interview in the project. (PDF 140 kb)
