# Supplementary material for: Similarities and differences between stakeholders’ opinions on using Health Technology Assessment (HTA) information across five European countries: results from the EQUIPT survey
Source: Health Res Policy Syst. 2016 May 26;14:38. doi: 10.1186/s12961-016-0110-7 (PMC4882811; doi:10.1186/s12961-016-0110-7)
Supplement: Additional file 1: — EQUIPT stakeholder interview survey questionnaire. Description of data: The questionnaire used in the stakeholder interview in the project. (PDF 140 kb) [file 12961_2016_110_MOESM1_ESM.pdf]

EQUIPT

European-study on Quantifying Utility of Investment in Protection from Tobacco

<u>The first page is to be filled in before the interview.</u>

1. Country: …………………………………………..

2. ID number: ………………………………………………………….

3. Date of the interview (dd-mm-yy): ……………………………………………………………….

4. Role of the interviewee:

a. Decision maker
b. Purchaser of services/pharma products
c. Professional, service provider
d. Evidence generator
e. Advocate of health promotion
f. Other, indicate:

5. Name of the interviewer (in capitals): ………………………………………………………………………………..

6. Duration of the interview: ………………………………………………………………………………

I hereby declare, that I handle the information collected securely, and provide it only for authorised person participating in the EQUIPT project. The data is anonymised before use.

…………………………………………..
Signature of the interviewer

Part A. Introduction

Hello, my name is ……….. from ………… We are participating in an international research project that investigates how the Tobacco Return On Investment Tool, developed by the National Institute for Health and Care Excellence (NICE) in the UK, can be adapted and transferred to different European countries, including [your country]. Hereafter, this tool is referred to as the "Tobacco ROI tool" for convenience.

The specific aims of the project are:

- to develop Tobacco ROI tools in four EU countries (Germany, Hungary, the Netherlands and Spain);
- to assess the needs for and ideas of using the Tobacco Return On Investment Tool to compare the results of ROI analysis across these countries;
- to transfer policy recommendations to other European countries not included in the sample.

In Part I. of the interview I will ask some general questions about setting priorities (what kind of information do you use when you make decisions on investments in tackling the scourge of tobacco?) related to tobacco control measures and smoking cessation interventions. In Part II. I will show you a presentation of the Tobacco ROI tool, and then I will ask you some questions about this tool.

We will tell you more about the Tobacco ROI tool in a minute.
To facilitate the best use of the ROI tool, we would ask for further input from you to fine tune the presentation of the tool and its results to ensure its relevance for practice.

Thank you for agreeing to provide a perspective. We appreciate your advice on how we can best shape this work so that it can provide greatest practical value to you. Our conversation should last approximately 40 minutes. I really appreciate your time and inputs to this process.

May we start the interview now? If you do not mind, I would tape-record it to save time. Is that okay?

Start the tape recorder and record the ID number

7. Can you briefly introduce yourself, your role and your responsibilities?

____

____

____

____

The Tobacco ROI tool basically calculates locally the return on investment for different packages of tobacco control measures. Please answer the upcoming question based on the information provided earlier. Here is your own copy of the questionnaire. Please answer the upcoming question based on the information provided earlier. We will provide you with more information about the tool in a minute

8. Could you please indicate on a scale from 1 to 7, 1 meaning “strongly disagree” and 7 meaning “strongly agree” to what extent you agree with the following statement: ‘’I have the intention to use an economic tool such as the Tobacco ROI for decision making/information gathering concerning tobacco control.’’ Please circle your answer as appropriate in the questionnaire.

Strongly Disagree						Strongly Agree	
1	2	3	4	5	6	7	?

9. Could you please indicate on a scale from 1 to 7 - 1 meaning “strongly disagree” and 7 meaning “strongly agree” to what extent you agree with the following statements? Please circle your answer in the column to the right.

	Strongly Disagree						Strongly Agree	
A. “Incremental costs” means how much the studied intervention itself costs more or less than the comparator intervention	1	2	3	4	5	6	7	?
B. When interventions in itself are cheap, they are always cost-effective compared to another intervention	1	2	3	4	5	6	7	?
C. “Willingness to pay” means how much a society is willing to pay for a quality adjusted life year or QALY	1	2	3	4	5	6	7	?
D. My intervention can be cost-effective compared to another intervention, even when its societal costs are higher than the regular care	1	2	3	4	5	6	7	?
E. In a health care payer perspective, indirect costs in full (such as productivity losses) are included	1	2	3	4	5	6	7	?

10. Could you please indicate on a scale from 1 to 7 - 1 meaning “strongly disagree” and 7 meaning “strongly agree” to what extent you agree with the following statements? Please circle your answer in the column to the right.

	Strongly Disagree						Strongly Agree	
A. The smoking epidemic is not severe in my country	1	2	3	4	5	6	7	?
B. Most smoking cessation interventions are effective	1	2	3	4	5	6	7	?
C. Most smoking cessation interventions are cost-effective	1	2	3	4	5	6	7	?
D. It is important to use smoking cessation interventions because smoking kills a lot of people	1	2	3	4	5	6	7	?
E. It is important to use smoking cessation interventions because smoking costs a lot for the society	1	2	3	4	5	6	7	?
F. It is unacceptable that we use smoking cessation interventions without knowing their efficacy	1	2	3	4	5	6	7	?
G. It is unacceptable that we use smoking cessation interventions without knowing their cost-effectiveness	1	2	3	4	5	6	7	?

Part B. Needs assessment

Now, let me show you a short video about the return on investment tool.

Show the video

11. Could you please indicate on a scale from 1 to 7 - 1 meaning "strongly disagree" and 7 meaning "strongly agree" to what extent you agree with the following statements? Please circle your answer in the column to the right.

If I were to use a tool such as the Tobacco ROI tool, it needs to give me:	Strongly Disagree						Strongly Agree	
A. Data on **prevalence** of smoking as reported in surveys and scientific studies	1	2	3	4	5	6	7	?
B. Data on **costs of smoking**	1	2	3	4	5	6	7	?
C. Data on **quality of life** as reported in surveys and scientific studies	1	2	3	4	5	6	7	?
D. Data on **mortality** due to smoking	1	2	3	4	5	6	7	?
E. **Effectiveness** of smoking cessation interventions (such as quit and relapse rate) as reported in scientific studies	1	2	3	4	5	6	7	?
F. **Cost-effectiveness** data comparing the cost of smoking cessation interventions with its health and wider benefits	1	2	3	4	5	6	7	?
G. **Budget impact** reflecting financial outcomes specifically at organisational level	1	2	3	4	5	6	7	?
H. Are there any other types of evidence that you consider important about the decision/information gathering on the implementation of tobacco control measures and smoking cessation interventions? ______ ______ ______								

12. Do you know about any other economic/financial models/tools of tobacco control measures/smoking cessation interventions (such as a decision tree or the WHO tobacco tax simulation model)?

a. No → Go to question 13
b. Yes

Could you please give one or more examples?

____

____

Part C. Tobacco ROI Tool

Now we would like to ask you some questions about the Tobacco ROI Tool just presented to you.
We are interested in your expectations or beliefs about the upcoming tool

13. Could you please indicate on a scale from 1 to 7 - 1 meaning “strongly disagree” and 7 meaning “strongly agree” to what extent you agree with the following statements? Please circle your answer in the column to the right.

	Strongly Disagree						Strongly Agree	
A. The Tobacco ROI tool has more advantages than disadvantages for me	1	2	3	4	5	6	7	?
What could be <u>advantages</u> for you of the Tobacco ROI tool? ***I belief that a tool such as the Tobacco ROI tool:***								
B. provides me with financial justification for my decision making/ information gathering	1	2	3	4	5	6	7	?
C. is easy to use	1	2	3	4	5	6	7	?
D. provides me with up-to-date information	1	2	3	4	5	6	7	?
E. provides relevant outcomes for my organisation	1	2	3	4	5	6	7	?
F. provides sufficient scientific support for decision making	1	2	3	4	5	6	7	?
G. provides added value to the information currently used	1	2	3	4	5	6	7	?
H. helps me decide which smoking cessation methods I should choose	1	2	3	4	5	6	7	?
I. integrates all relevant information available to calculate cost-effectiveness	1	2	3	4	5	6	7	?
J. can calculate the cost-effectiveness outcomes over different periods such as 2 years, 5 years, 10 years, and lifetime	1	2	3	4	5	6	7	?
K. can assess cost-effectiveness of potential new interventions	1	2	3	4	5	6	7	?
L. helps increase efficiency in service delivery	1	2	3	4	5	6	7	?
M. What other advantages would a tool such as the Tobacco ROI tool have for you? ____________ ____________ ____________ ____________								

What could be <u>disadvantages</u> for you of the Tobacco ROI tool? ***I belief that a tool such as the Tobacco ROI tool:***	Strongly Disagree						Strongly Agree	
N. is too time consuming to use	1	2	3	4	5	6	7	?
O. is not useful for my organisation	1	2	3	4	5	6	7	?
P. requires too much data input	1	2	3	4	5	6	7	?
Q. is too complex to work with	1	2	3	4	5	6	7	?
R. is not compatible with the way we work in our organisation	1	2	3	4	5	6	7	?
S. provides unreliable outcomes	1	2	3	4	5	6	7	?
T. does not provide insight on how the results are calculated	1	2	3	4	5	6	7	?
U. results into outcomes that are too broad	1	2	3	4	5	6	7	?
V. is not needed for my daily work	1	2	3	4	5	6	7	?
W. What other disadvantages would a tool such as the Tobacco ROI tool have for you? ____ ____ ____ ____								

14. Could you please indicate on a scale from 1 to 7 - 1 meaning “strongly disagree” and 7 meaning “strongly agree” to what extent you agree with the following statements? Please circle your answer in the column to the right.

	Strongly Disagree						Strongly Agree	
A. My boss will support me in using a tool such as the Tobacco ROI tool in decision making/information gathering	1	2	3	4	5	6	7	?
B. My other colleagues will support me in using a tool such as the Tobacco ROI tool	1	2	3	4	5	6	7	?
C. My organisation will support me in using a tool such as the Tobacco ROI tool in decision making/information gathering	1	2	3	4	5	6	7	?
D. Reimbursement agencies (e.g. health insurances) will support me in using a tool such as the Tobacco ROI tool in decision making/information gathering	1	2	3	4	5	6	7	?
E. My ministry of health will support me in using a tool such as the Tobacco ROI tool in decision making/information gathering	1	2	3	4	5	6	7	?
F. Health professionals (i.e. GPs, medical specialists) will support me in using a tool such as the Tobacco ROI tool in decision making/information gathering	1	2	3	4	5	6	7	?
G. Who else would support you in using the Tobacco ROI tool?								

___ ___ ___ ___								
H. I would encounter resistance using the Tobacco ROI tool	1	2	3	4	5	6	7	?
I. Which organisations or colleagues would not support you to use the Tobacco ROI tool?<br>___<br>___								

**How confident are you about using the Tobacco ROI tool?**<br>**I am confident that:**	Strongly Disagree						Strongly Agree	
J. I will be able to use the ROI tool	1	2	3	4	5	6	7	?
K. I will be able to use the ROI tool when I need to enter the information on smoking cessation effects of programs myself	1	2	3	4	5	6	7	?
L. I will be able to understand the ROI tool when the data is given in a list of figures	1	2	3	4	5	6	7	?
M. I will be able to use the ROI tool even if my organisation does not support me	1	2	3	4	5	6	7	?
N. I will be able to use the ROI tool without help from others	1	2	3	4	5	6	7	?
O. Persons such as myself in my organisation will be able to use the ROI tool	1	2	3	4	5	6	7	?
P. I will be able to interpret the output of the ROI tool	1	2	3	4	5	6	7	?
Q. I will be able to use the tool with limited knowledge about health economics	1	2	3	4	5	6	7	?
R. I will be able to use the ROI tool without technical support	1	2	3	4	5	6	7	?
S. I will be able to use the ROI tool without a manual	1	2	3	4	5	6	7	?
T. I will be able to use the ROI tool without a website that explains how the ROI tool works	1	2	3	4	5	6	7	?
U. What other difficulties would using a tool such as the Tobacco ROI tool have for you?<br>___<br>___<br>___<br>___								

15. Could you please indicate on a scale from 1 to 7 - 1 meaning “strongly disagree” and 7 meaning “strongly agree” to what extent you agree with the following statements? Please circle your answer in the column to the right.

	Strongly Disagree						Strongly Agree	
A. I have the intention to use a tool such as the Tobacco ROI tool	1	2	3	4	5	6	7	?
B. I have the intention to use a tool such as the Tobacco ROI tool within the next month	1	2	3	4	5	6	7	?
C. I have the intention to use a tool such as the Tobacco ROI tool within the next 6 months	1	2	3	4	5	6	7	?
D. I have the intention to use a tool such as the Tobacco ROI tool within the next year	1	2	3	4	5	6	7	?
E. I would like to have more information about the Tobacco ROI tool	1	2	3	4	5	6	7	?

Part D. Availability of tobacco control measures & smoking cessation interventions

16. Please read this table! Would you tell me whether these tobacco control measures and smoking cessation interventions are available in ………? Could you also please indicate on a scale from 1 to 3 - 1 meaning "not important" and 3 meaning "important" - to what extent you think the following interventions are considered important in addressing smoking behaviour?

Note that the table proceeds on the next page.

Category	Name of intervention	Availability			Importance			
Pharmacological	Nicotine replacement therapy	Yes	No	?	1	2	3	?
	Bupropion	Yes	No	?	1	2	3	?
	Varinicline	Yes	No	?	1	2	3	?
Behavioural	Brief advice on smoking cessation given during one GP consultation	Yes	No	?	1	2	3	?
	Advice on smoking cessation given according to the 5-step protocol (minimal intervention)	Yes	No	?	1	2	3	?
	Community pharmacy-based services	Yes	No	?	1	2	3	?
	Computer tailored programs	Yes	No	?	1	2	3	?
	Internet based interventions	Yes	No	?	1	2	3	?
	Group counselling by specially trained professionals	Yes	No	?	1	2	3	?
	Individual counselling by specially trained professionals	Yes	No	?	1	2	3	?
	Telephone counselling	Yes	No	?	1	2	3	?
	Self-help manuals	Yes	No	?	1	2	3	?
	Mobile phone-based interventions	Yes	No	?	1	2	3	?
	Stage-based interventions	Yes	No	?	1	2	3	?
Combined	Individual counselling by specially trained professionals with	Yes	No	?	1	2	3	?

	medication (e.g. NRT or bupropion)								
	Group counselling by specially trained professionals with medication (e.g. NRT or bupropion)	Yes	No	?	1	2	3	?
	Brief advice by GP and medication	Yes	No	?	1	2	3	?
Nonconventional	Acupuncture	Yes	No	?	1	2	3	?
	Aromatherapy	Yes	No	?	1	2	3	?
	Herbs	Yes	No	?	1	2	3	?
	Smokeless tobacco	Yes	No	?	1	2	3	?
	Hypnosis-based interventions	Yes	No	?	1	2	3	?
	Magnetic resonance therapy	Yes	No	?	1	2	3	?
	Homeopathy	Yes	No	?	1	2	3	?
Preventative	Advertising restrictions/bans	Yes	No	?	1	2	3	?
	Product labelling and information/ Health warnings on tobacco products	Yes	No	?	1	2	3	?
	Restrictions on sales to minors	Yes	No	?	1	2	3	?
	Restrictions on smoking in workplaces and public places	Yes	No	?	1	2	3	?
	Mass media campaigns	Yes	No	?	1	2	3	?
	Tax increase	Yes	No	?	1	2	3	?

17. What would you like to get out of being involved in this research project for you and your organisation?

____

____

____

18. The current version of the ROI model includes the following smoking-related diseases: lung cancer, coronary heart disease, chronic obstructive pulmonary disease, myocardial infarction, and stroke. Are there any other smoking related diseases that you would include in the ROI tool?

____

____

____

19. Comments about the interview:

____

____

____

____
